# The effect of right ventricular compression on cardiac function in pediatric pectus excavatum

**DOI:** 10.1186/1532-429X-16-S1-P250

**Published:** 2014-01-16

**Authors:** Animesh Tandon, Daniel Wallihan, Adam M Lubert, Michael D Taylor

**Affiliations:** 1 Heart Institute, Cincinnati Children's Hospital Medical Center, Cincinnati, Ohio, USA

## Background

Pectus excavatum can cause right ventricular (RV) dysfunction due to extrinsic compression. Assessment of ventricular function by echocardiography is often suboptimal due to technical limitations. We utilized cardiac magnetic resonance imaging (CMR) to assess pectus severity and RV systolic function in relation to the site of RV compression in a large pediatric pectus cohort.

## Methods

All CMR studies performed for clinical evaluation of pediatric pectus excavatum at our institution were retrospectively reviewed. CMR analysis included evaluation of Haller index, left ventricular (LV) and RV ejection fractions (EF), and indexed LV and RV end-diastolic and end-systolic volumes. The site of maximal compression of the right ventricle (no compression, atrioventricular (AV) groove, or free wall) was assessed. The relationships of age, BSA, Haller index, and ln(Haller) to LVEF, RVEF, and indexed RV end-diastolic volume were assessed using linear regression. The relationships of patient gender and RV compression site to LVEF, RVEF, and indexed RV end-diastolic volume were evaluated using ANOVA with post-hoc Tukey comparisons.

## Results

We analyzed CMR studies from 197 patients with pectus excavatum (163 males, 34 females, age 2.67-24.9 yrs). 28 patients (14.2%) had RVEF<50%. 75 patients had no RV compression, 104 had compression of the RV free wall, and 18 had compression of the AV groove. The Haller index, ln(Haller), and age did not show linear relationships to LVEF, RVEF, or indexed RV end-diastolic volume. The LVEF was significantly lower in patients with RV compression (65.9% vs. 63.1%, p = 0.0002), although still in the normal range. RVEF varied significantly between RV compression groups (no compression 58.3%, free wall 54.7%, AV groove 51.7%, all p < 0.04). The indexed RV end-diastolic volume was increased in patients with RV compression (96.5 mL/m2) compared to those without compression (89.3 mL/m2, p = 0.0079).

## Conclusions

CMR imaging is a reliable method to measure pectus excavatum severity, associated sternal abnormalities, and cardiac function in pediatric patients in a single imaging study, obviating the need for echocardiography and computed tomography. RV dysfunction is common, even in pediatric pectus patients. The RVEF varies significantly based on the site of RV compression; however, the Haller index does not predict the severity of RV systolic dysfunction.

## Funding

Cincinnati Children's Heart Institute Research Core.

**Table 1:** Relationships between LVEF, RVEF, and RV end-diastolic volume to geometric markers

	Range or n	LVEF (%); p-value	RVEF (%); p-value	**Indexed RV end-diastolic volume (mL/m2); p-value**a
Age	2.67-24.9 yrs	NS	NS	NS

BSA	0.62-2.20 m2	NS	NS	NS

Haller	2.1-39	NS	NS	NS

ln(Haller)	0.74-3.67	NS	NS	NS

Male vs. female	163 vs. 34	NS	NS	95.5 vs. 85.4; p = 0.0039

RV compression, no vs. yes	75 vs. 122	65.9 vs. 63.1; p = 0.0002	58.3 vs. 54.3; p < 0.0001	89.3 vs. 96.5; p = 0.0079

RV compression: free wall vs. AV groove	104 vs. 18	63.2 vs. 62.2; p = NS	54.7 vs. 51.6; p = 0.0413	95.1 vs. 104.5; p = NS

Range, n, or mean values are shown. For hypothesis testing, p-values of < 0.05 were considered significant. NS = not significant.

